# Supplementary material for: Temporal inhibition of chromatin looping and enhancer accessibility during neuronal remodeling
Source: Nat Commun. 2021 Nov 4;12:6366. doi: 10.1038/s41467-021-26628-7 (PMC8568962; doi:10.1038/s41467-021-26628-7)
Supplement: Supplementary file 1 — Supplementary Information [file 41467_2021_26628_MOESM1_ESM.pdf]

SUPPLEMENTARY INFORMATION

Temporal inhibition of chromatin looping and enhancer accessibility during neuronal remodeling

Dahong Chen, Catherine E. McManus, Behram Radmanesh, Leah H. Matzat, and Elissa P. Lei

Fig. S1

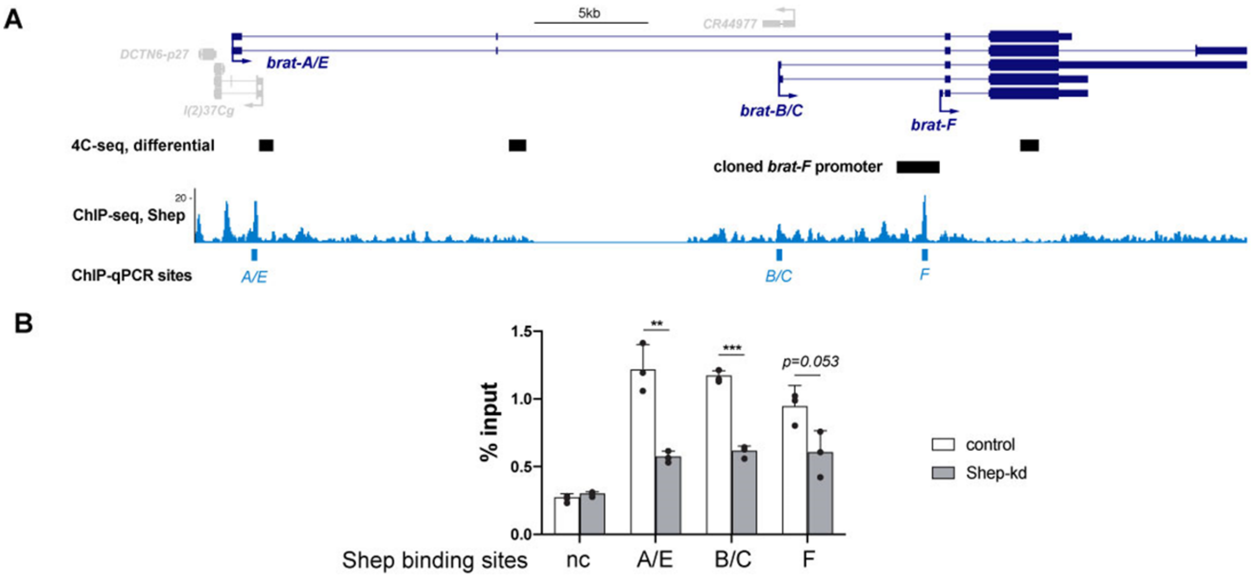

Fig. S1. Validation of Shep chromatin association in BG3 cells. BG3 cells treated with GFP or Shep dsRNA were used for ChIP-qPCR quantification of Shep binding near three *brat* isoform promoters. Significant or marginally significant reduction of Shep binding was observed for all three loci upon Shep depletion. **A)** Screenshot and **B)** directed ChIP-qPCR quantification of three biological replicates. Data are presented as mean values + SEM and Student's *t* test was performed for each independent locus with **p<0.01, ***p<0.001. The exact p values are 3.8e-5 and 4e-3 for *brat-B/C* and *brat-A/E* loci, respectively.

Fig. S2.

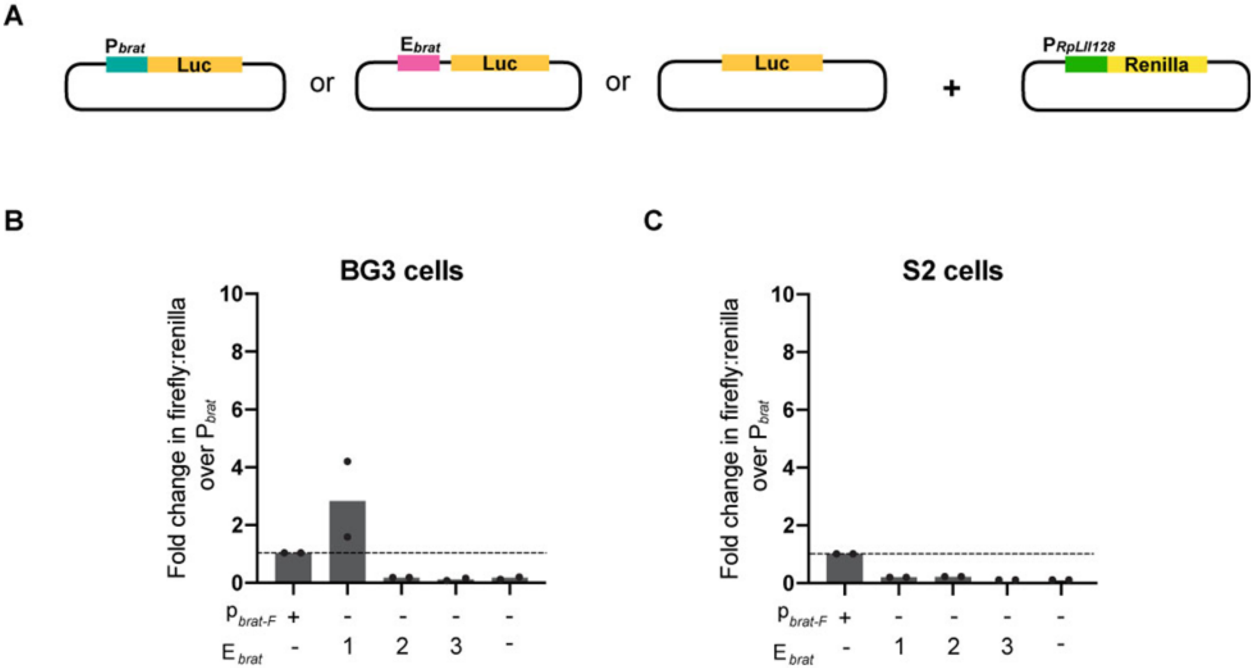

Fig. S2. Shep does not repress activity of the region 1 enhancer or any *brat* promoters. (A) Control constructs with luciferase cloned downstream of the *brat-F* promoter or individual enhancer candidates alone were co-transfected with the Renilla control construct. **(B)** Fold change over *brat-F* promoter alone driven expression of Renilla-normalized luciferase activity in BG3 cells. None of the enhancer candidates alone drive stronger luciferase than the *brat-F* promoter in BG3 cells. Experiments were performed with n=2 biologically independent samples. **(C)** Identical transfections as in (B) in S2 cells. Experiments were performed with n=2 biologically independent samples.

Fig. S3.

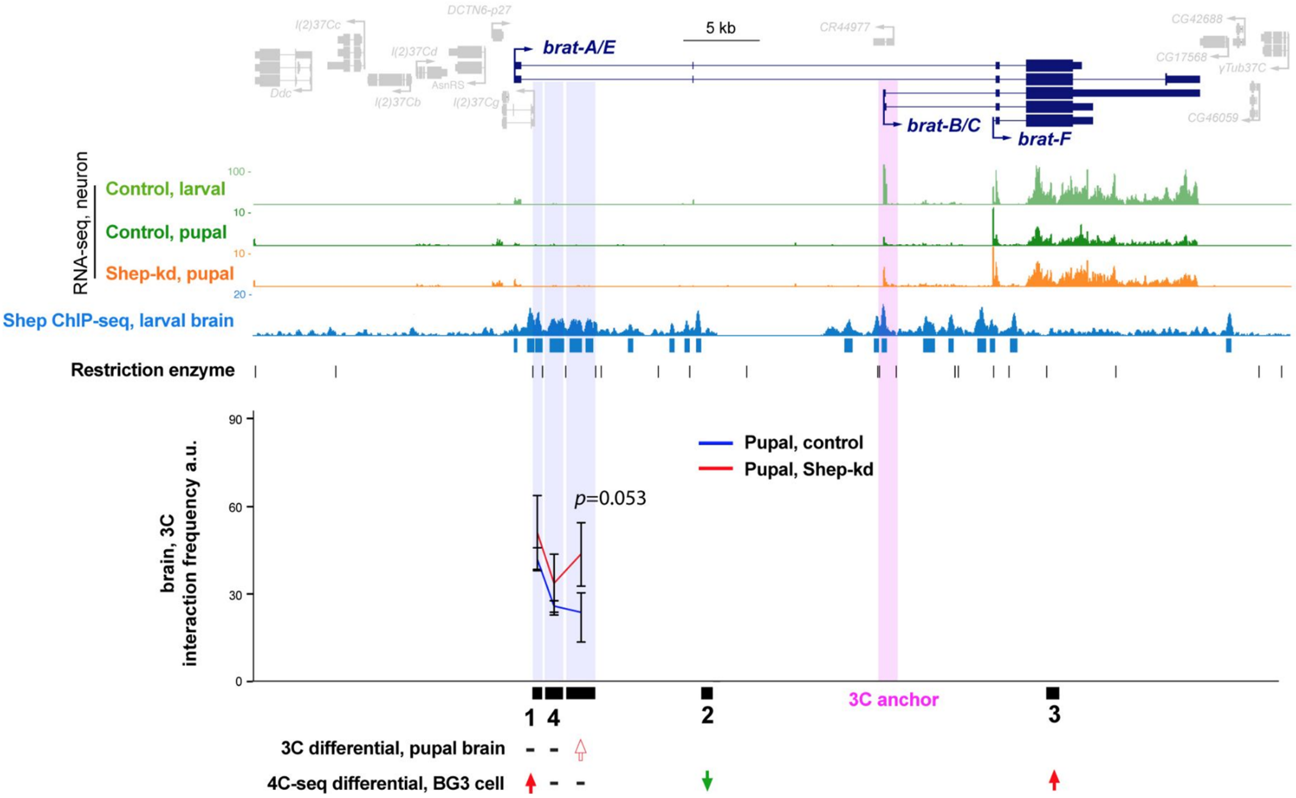

Fig. S3. Pupal brain 3C assays with the *brat-B/C* promoter as the anchor. Three biological replicates were included for each genotype. Enhancer 1 and enhancer 4 do not show changed interaction frequencies with the *brat-B/C* promoter. The 3' region shows a marginally significant increase of interaction frequency. Note difference in 3C scale compared to Figure 3. Experiments were performed with n=3 biologically independent samples, and data are presented as mean values +/- SEM. Two-tailed Student's *t* test was performed.

Fig. S4.

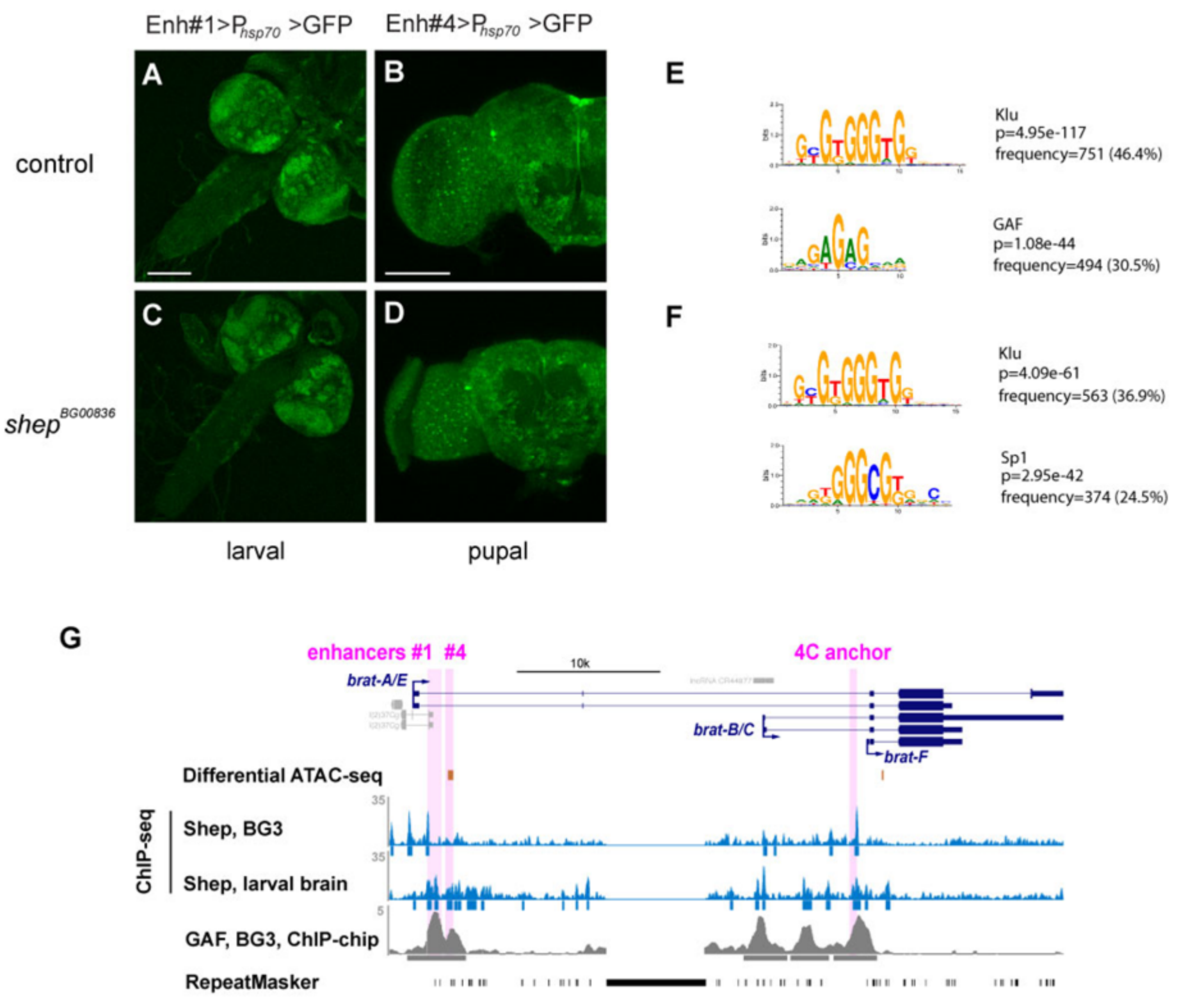

Fig. S4. Region 1 and 4 enhancer activities and accessibility are unchanged in Shep-depleted pupal brains and neurons. (A-D) GFP expression driven by region 1 or 4 directly upstream of the *hsp70* minimal promoter in larval or pupal brains, respectively, in control or strong loss-of-function *shep* mutant brains. Experiment was performed once using multiple individuals for each genotype. Scale bars: 100 µm. **(E)** Top motifs identified by motif enrichment analyses using algorithms AME and STREME of H3K4me1-labeled Shep-inhibited accessible regions in pupal neurons. Frequency indicates the proportion of Shep-inhibited enhancers that harbor respective motifs. Unadjusted *p* values are derived from AME. **(F)** Motifs identified for enhancers of Shep-promoted accessibility. Unadjusted *p* values are derived form AME. **(G)** GAF associates with regulatory elements and promoters of *brat* in BG3 cells.

Fig. S5.

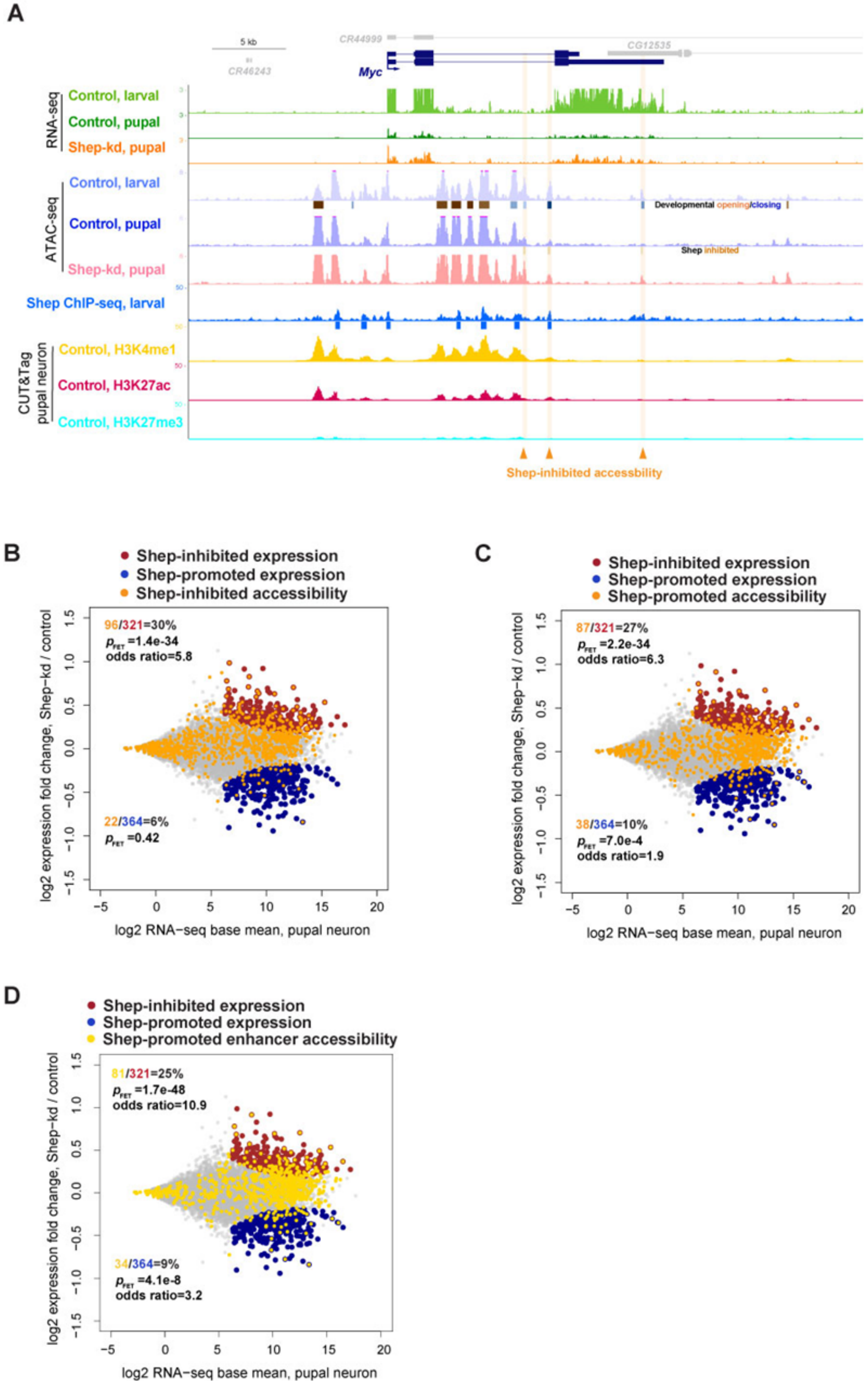
A
5 kb
CR44999
CR46243
CG12535
Myc
RNA-seq
Control, larval
Control, pupal
Shep-kd, pupal
ATAC-seq
Control, larval
Control, pupal
Shep-kd, pupal
Developmental opening/closing
Shep inhibited
Shep ChIP-seq, larval
CUT&Tag pupal neuron
Control, H3K4me1
Control, H3K27ac
Control, H3K27me3
Shep-inhibited accessbility
B
Shep-inhibited expression
Shep-promoted expression
Shep-inhibited accessibility
96/321=30%
p_{FET} =1.4e-34
odds ratio=5.8
22/364=6%
p_{FET} =0.42
log2 expression fold change, Shep−kd / control
log2 RNA−seq base mean, pupal neuron
C
Shep-inhibited expression
Shep-promoted expression
Shep-promoted accessibility
87/321=27%
p_{FET} =2.2e-34
odds ratio=6.3
38/364=10%
p_{FET} =7.0e-4
odds ratio=1.9
log2 expression fold change, Shep−kd / control
log2 RNA−seq base mean, pupal neuron
D
Shep-inhibited expression
Shep-promoted expression
Shep-promoted enhancer accessibility
81/321=25%
p_{FET} =1.7e-48
odds ratio=10.9
34/364=9%
p_{FET} =4.1e-8
odds ratio=3.2
log2 expression fold change, Shep−kd / control
log2 RNA−seq base mean, pupal neuron

Fig. S5. Genome-wide association between Shep-regulated expression and accessibility. (A) Pupal-specific Shep inhibition of *Myc* expression (orange track, p=5.6e-3, fold change=1.5) and accessibility of H3K4me1-labeled regions (orange shading, FDR=0.06, 0.05, and 0.03, respectively; fold change=1.3, 1.3 and 1.5, respectively). FDR was calculated by edgeR (see Methods). **(B)** Overlap between Shep-dependent expression and Shep-inhibited accessibility in pupal neurons. Genes inhibited for expression by Shep are enriched for Shep-inhibited accessible regions (red and orange). The p values of two-tailed FET without adjustment are reported for indicated colored groups. **(C)** Similar overlap to (B) but for Shep-promoted accessibility. **(D)** Similar overlap to (C) but restricted to Shep-promoted enhancer accessibility. Similar analysis for Shep-inhibited enhancer accessibility is shown in Figure 4E.
