# Supplementary material for: Temporal inhibition of chromatin looping and enhancer accessibility during neuronal remodeling
Source: Nat Commun. 2021 Nov 4;12:6366. doi: 10.1038/s41467-021-26628-7 (PMC8568962; doi:10.1038/s41467-021-26628-7)
Supplement: Supplementary file 3 — Description for Additional Supplementary Files [file 41467_2021_26628_MOESM3_ESM.docx]

File Name: Supplementary Data 1

Description: This supplementary file includes sequences of primers used to perform RT-qPCR, ChIP-qPCR, 3C-Taqman-qPCR, 4C-seq and luciferase-related cloning assays. This file also contains plasmid sequences used to delete the region 4 enhancer and primers to validate the successful deletion. Genomic coordinates of the Shep-regulated ATAC-seq peaks in larval and pupal neurons are also included.
