# Supplementary material for: Temporal inhibition of chromatin looping and enhancer accessibility during neuronal remodeling
Source: Nat Commun. 2021 Nov 4;12:6366. doi: 10.1038/s41467-021-26628-7 (PMC8568962; doi:10.1038/s41467-021-26628-7)
Supplement: Supplementary file 5 — Reporting Summary [file 41467_2021_26628_MOESM5_ESM.pdf]

nature research

Corresponding author(s): Elissa P. Lei

Last updated by author(s): Sep 21, 2021

Reporting Summary

Nature Research wishes to improve the reproducibility of the work that we publish. This form provides structure for consistency and transparency in reporting. For further information on Nature Research policies, see our Editorial Policies and the Editorial Policy Checklist.

Statistics

For all statistical analyses, confirm that the following items are present in the figure legend, table legend, main text, or Methods section.

n/a	Confirmed	
☐	☒	The exact sample size (*n*) for each experimental group/condition, given as a discrete number and unit of measurement
☐	☒	A statement on whether measurements were taken from distinct samples or whether the same sample was measured repeatedly
☐	☒	The statistical test(s) used AND whether they are one- or two-sided<br>*Only common tests should be described solely by name; describe more complex techniques in the Methods section.*
☒	☐	A description of all covariates tested
☒	☐	A description of any assumptions or corrections, such as tests of normality and adjustment for multiple comparisons
☒	☐	A full description of the statistical parameters including central tendency (e.g. means) or other basic estimates (e.g. regression coefficient) AND variation (e.g. standard deviation) or associated estimates of uncertainty (e.g. confidence intervals)
☒	☐	For null hypothesis testing, the test statistic (e.g. *F*, *t*, *r*) with confidence intervals, effect sizes, degrees of freedom and *P* value noted<br>*Give P values as exact values whenever suitable.*
☒	☐	For Bayesian analysis, information on the choice of priors and Markov chain Monte Carlo settings
☒	☐	For hierarchical and complex designs, identification of the appropriate level for tests and full reporting of outcomes
☒	☐	Estimates of effect sizes (e.g. Cohen's *d*, Pearson's *r*), indicating how they were calculated

Our web collection on statistics for biologists contains articles on many of the points above.

Software and code

Policy information about availability of computer code

Data collection	no software was used
Data analysis	DiffBind v2.6.6, bowtie2 v2.3.5, Samtools v1.9, and FourCSeq v1.18.0 were used to analyze 4C-seq data. cutadapt v2.3, bowtie2 v2.3.5, samtools v1.9, macs2 v2.2.6 and DiffBind were used to analyze ATAC-seq, ChIP-seq and CUT&Tag data.

For manuscripts utilizing custom algorithms or software that are central to the research but not yet described in published literature, software must be made available to editors and reviewers. We strongly encourage code deposition in a community repository (e.g. GitHub). See the Nature Research guidelines for submitting code & software for further information.

Data

Policy information about availability of data

All manuscripts must include a data availability statement. This statement should provide the following information, where applicable:

- Accession codes, unique identifiers, or web links for publicly available datasets
- A list of figures that have associated raw data
- A description of any restrictions on data availability

The 4C-seq, ATAC-seq, CUT&Tag, and ChIP-seq data have been deposited in the Gene Expression Omnibus database under accession number GSE154645. All figures are associated with these data.

Field-specific reporting

Please select the one below that is the best fit for your research. If you are not sure, read the appropriate sections before making your selection.

[x] Life sciences [ ] Behavioural & social sciences [ ] Ecological, evolutionary & environmental sciences

For a reference copy of the document with all sections, see nature.com/documents/nr-reporting-summary-flat.pdf

Life sciences study design

All studies must disclose on these points even when the disclosure is negative.

Sample size	All quantitative tests were performed in biological triplicates except where indicated.
Data exclusions	No data were excluded.
Replication	All replicates are independent, biological replicates that show consistent results. Replicate samples were collected on different days independently. Assays and sequencing libraries were performed and generated on different days as independent replications.
Randomization	Samples were collected and grouped by stages, treatments or genotypes.
Blinding	Blinding was not included due to intended knockdown treatments but all differential analyses and calling were performed with statistical methods without artificial selection.

Reporting for specific materials, systems and methods

We require information from authors about some types of materials, experimental systems and methods used in many studies. Here, indicate whether each material, system or method listed is relevant to your study. If you are not sure if a list item applies to your research, read the appropriate section before selecting a response.

Materials & experimental systems

n/a	Involved in the study	
[ ]	[x]	Antibodies
[ ]	[x]	Eukaryotic cell lines
[x]	[ ]	Palaeontology and archaeology
[ ]	[x]	Animals and other organisms
[x]	[ ]	Human research participants
[x]	[ ]	Clinical data
[x]	[ ]	Dual use research of concern

Methods

n/a	Involved in the study	
[ ]	[x]	ChIP-seq
[x]	[ ]	Flow cytometry
[x]	[ ]	MRI-based neuroimaging

Antibodies

Antibodies used

GFP antibody (ThermoFisher A10262), H3K27me3 (Cell Signaling Technology 9733), H3K4me1 (Abcam ab8895), and H3K27ac (Abcam ab4729). Generation of the guinea pig anti-Shep (Matzat et al., PLoS Gen, 2012) was designed by Leah Matzat at the NIDDK.

Validation

ThermoFisher A10262: Immunofluorescent analysis of GFP Tag was performed using H3-GFP construct transfected in HEK-293E cells, Published species: Fruit fly, Mouse

Cell Signaling Technology 9733:
Application Key: WB-Western Blot IP-Immunoprecipitation IHC-Immunohistochemistry ChIP-Chromatin Immunoprecipitation IF-Immunofluorescence F-Flow Cytometry E-P-ELISA-Peptide
Species Cross-Reactivity Key: H-Human M-Mouse R-Rat Hm-Hamster Mk-Monkey Vir-Virus Mi-Mink C-Chicken Dm-D. melanogaster

Abcam ab8895:
Tested applications Suitable for: ICC, ChIP, WB, IHC-P
Species reactivity Reacts with: Mouse, Rat, Cow, Human, Predicted to work with: Pig, Saccharomyces cerevisiae, Tetrahymena, Xenopus laevis, Drosophila melanogaster,

AAbcam ab4729
Tested applications Suitable for: ICC, ChIP, WB, IHC-P
Species reactivity Reacts with: Mouse, Rat, Cow, Human, Predicted to work with: Pig, Saccharomyces cerevisiae, Tetrahymena, Xenopus laevis, Drosophila melanogaster,

Anti-Shep: Furthermore, four different homozygous P-element insertions result in loss of Shep protein, two greatly reducing all

isoforms (Figure 3B, lanes 2–3) and two eliminating isoform A (lanes 7–8)., Matzat et al. PLoS Gen, 2012

Eukaryotic cell lines

Policy information about cell lines

Cell line source(s)	DGRC, BG3-c2 cells, S2 cells
Authentication	Cells show close molecular features, morphology, behavior, growth rate and medium requirement as the commercial authorization.
Mycoplasma contamination	Not tested
Commonly misidentified lines (See ICLAC register)	No commonly misidentified cell lines were used in the study

Animals and other organisms

Policy information about studies involving animals; ARRIVE guidelines recommended for reporting animal research

Laboratory animals	Drosophila melanogaster stocks and crosses were grown on standard cornmeal-yeast-agarose medium at 25°C and only female animals were used. Fly strains used include elav-Gal4 (P{GawB}elavC155; FBst0000458), UAS-Dcr-2 (w1118; P{UAS-Dcr-2.D}2; FBst0024650) and UAS-shep-RNAi (w1118; P{GD5125}v37863; FBst0462204). Only female third instar larvae or 82h pupae were used for experiments
Wild animals	did not involve wild animals.
Field-collected samples	did not involve field samples.
Ethics oversight	No vertebrates were used so no ethics protocol was involved.

Note that full information on the approval of the study protocol must also be provided in the manuscript.

ChIP-seq

Data deposition

☒ Confirm that both raw and final processed data have been deposited in a public database such as GEO.

☒ Confirm that you have deposited or provided access to graph files (e.g. BED files) for the called peaks.

Data access links<br>*May remain private before publication.*	GSE154645
Files in database submission	LM49_input-brains.trim.unique.nodups_CPMnormalized.bw<br>LM50_shep-brains.trim.unique.nodups_CPMnormalized.bw<br>LM50_shep-brains.fastq.gz<br>LM49_input-brains.fastq.gz<br>https://hpc.nih.gov/~chend8/2021/ChIP/Ryan_ChIP/larva_brain/MACS2_peaks/q0.2/LM50_shep-brains_peaks.narrowPeak
Genome browser session (e.g. UCSC)	no longer applicable

Methodology

Replicates	one replicate to profile Shep binding profile
Sequencing depth	SE sequencing fragment = 124bp, raw reads = 12.2 million, uniquely mapped reads = 8.3 million
Antibodies	guinea pig anti-Shep (Matzat et al., PLoS Gen, 2012)
Peak calling parameters	macs2 callpeak -t {input.chip} -c {input.control} -f BAM -g dm -n {wildcards.sample} -B -q 0.2
Data quality	ChIP-seq peaks are confirmed by ChIP-qPCR
Software	cutadapt v2.3, bowtie2 v2.3.5, samtools v1.9, macs2 v2.2.6

April 2020
